# *European Journal of Medical Research* reviewer acknowledgement 2013

**DOI:** 10.1186/2047-783X-19-4

**Published:** 2014-02-05

**Authors:** Dieter Häussinger

**Affiliations:** 1 University of Düsseldorf, Düsseldorf, Germany

## Abstract

**Contributing reviewers:**

The editor of *European Journal of Medical Research* would like to thank all our reviewers who have contributed to the journal in Volume 18 (2013).

## 

Ahmed Abdel Razek

Egypt

Simon Abi Aad

United States Of America

Adewale Adeniran

United States Of America

Vikram Agarwal

United States Of America

Metin Akbulut

Turkey

Ovunc Akdemir

Turkey

Feras Al-Shahrabani

Germany

Jorge Amil Dias

Portugal

Martin Angele

Germany

Victor Asensi

Spain

Matthias Aurich

Germany

Eric Bader

United States Of America

Mert Ozan Bahtiyar

United States Of America

Giuseppe Banfi

Italy

Orlin Belyaev

Germany

Alexandre Benjo

United States Of America

Christoph Berger

Switzerland

Uwe Bergmann

Germany

Sabine Bischoff

Germany

Arjen Blom

Netherlands

Edwin Boelke

Germany

Yerko Borghero

Christmas Island

Joaquim Bosch-Barrera

Spain

Michael Brauckhoff

Norway

Antonio Brehm

Portugal

Juergen Brunner

Austria

Francisco Yuri Bulcao Macedo

United States Of America

Franco Maria Buonaguro

Italy

Miguel Cabada

Peru

Aras Emre Canda

Turkey

Bin Cao

China

Antonio Celada

Spain

Jack Chen

United States Of America

Wei Chen

China

William Cho

Hong Kong

George Chrousos

Greece

Peter Conzen

Germany

Miguel Cruz

Mexico

Ana Cuesta Fernandez

United States Of America

Long Cui

China

Yubao Cui

China

Bruno D'agostino

Italy

Jane Daniels

United Kingdom

Kaid Darwiche

Germany

Sarwa Darwish Murad

Netherlands

D Das

Azerbaijan

Ravi Dasu

United States Of America

John E. Davies

Canada

Zhong-Liang Deng

China

Ilhem Diboun

American Samoa

Markus K. Diener

Germany

Qiang Ding

United States Of America

Amolkumar Diwan

India

Martine Docx

Belgium

Pere Domingo

Spain

Kathrin Doppler

Germany

Dimitrios Dragoumis

Greece

Koen Dreijerink

Netherlands

Peter Dungel

Austria

Ali El Saman

Egypt

Hedley Emsley

United Kingdom

Claudius Falch

Germany

Zuoxu Fan

China

David Faraoni

Belgium

Nicolas Feltgen

Germany

Xavier Forceville

France

Massimo Franchini

Italy

Randall Franz

United States Of America

Jose Humberto Fregnani

Brazil

Mayuko Furuta

Japan

Grammati Galaktidou

Greece

Andrea Galosi

Italy

Apar Kishor Ganti

United States Of America

Jose M Gatell

Spain

Osama Gheith

Egypt

Olivier Gires

Germany

Nicola Giuliani

Italy

Jorg Glatzle

Germany

Michael Gliem

Germany

Tom Glomsaker

Norway

Ines Gockel

Germany

Adams Grant

Germany

Johannes Grillari

Austria

Flávia Guarnier

Brazil

Brian J Haas

Antarctica

William Hau

Hong Kong

Pingan He

China

My Helms

United States Of America

Gerd Heusch

Germany

Martin Hoenigl

Austria

Katrin Hoffmann

Germany

Sakae Homma

Japan

Hanson Hon

China

Dawei Huang

Armenia

Wei Huang

China

Yi Huang

China

Arturo Huerta

Spain

Bernd Hüneke

Germany

Kenneth Iczkowski

United States Of America

Fumiaki Isohashi

Japan

John Janowskiq

United Kingdom

Jessica Jennings

United States Of America

Dadi Jin

China

Zheng Junch

China

Ayeesha Kamran Kamal

Pakistan

Theodoros Karaiskos

Greece

Nikolaos Katsikogiannis

Greece

Naureen Keric

Germany

Asma Khalil

United Kingdom

Salman Khan

Korea South

Tae Hyun Kim

Korea South

Atsushi Kitani

United States Of America

Hjh Kjk

Bahrain

Piotr Korczyski

Poland

Lydia Kriegl

Aruba

Robert Kubina

Poland

Keiichi Kubota

Japan

David Lappin

United Kingdom

Sergio Larach

United States Of America

Joao Carlos Leal

Brazil

Kuo Ann Lee

United Kingdom

Jun Li

Germany

Li Li

China

Yan Li

United States Of America

Zhi Li

China

Carol Liang

United States Of America

Dimitrios Linos

Greece

Jun Liu

United States Of America

Josep M Llibre

Spain

Simon Lo

United States Of America

Guangming Lu

China

Guobing Lu

United Kingdom

Xianghang Luo

China

Lin Ma

China

Paolo Macchiarini

Sweden

John Macleod

Canada

Bernhard Maisch

Germany

Richard Malthaner

Canada

Christos Maniotis

Greece

Deepa Manwani

United States Of America

John C. Marioni

American Samoa

James Martin

Canada

Diego Maselli

United States Of America

Praveen Mathur

United States Of America

Catalina Matiz

United States Of America

Christiane Matuschek

Germany

Roberta Mazzucchelli

Italy

Philip Mcternan

United Kingdom

Golbarg Mehrpoor

Iran

Ashish Mehta

United States Of America

Stephan Meller

Germany

Christian Meyer

Germany

Melissa Meyer Zu Hoerste

United States Of America

Radu Mihai

United Kingdom

Nicola Modugno

Italy

Pooi-Ling Mok

Malaysia

Fernando Moraes

Brazil

George Mouzopoulos

Greece

Maria Aparecida Nagai

Brazil

Jose Najera

United States Of America

Eiji Nemoto

Japan

Richard Niles

United States Of America

Michiru Nishita

Japan

Sadat Noori

Iran

Mark Oette

Germany

Boguslaw Okopien

Poland

Serdar Ozbas

Turkey

Samantha P

American Samoa

Vassilios Papalois

United Kingdom

Roger Paredes

Spain

Tobias Peikert

United States Of America

Jirun Peng

China

Francisco Pérez Roldán

Spain

Frank Pfeffer

Norway

Claudia Piccoli

Italy

Andres Pineda

United States Of America

Michael Pinkawa

Germany

Matthias Pinter

Austria

Beyzagul Polat

Turkey

Ibrahim Polat

Turkey

Guixing Qiu

China

Qiang Qu

China

Claudia Rauh

Germany

Guosheng Ren

China

Tyvin A Rich

American Samoa

Robert Rieben

Switzerland

Maria Ripoli

Italy

Marianne Rohrbach

Switzerland

Axel Rolle

Germany

Richard Roman

United States Of America

Guanghua Rong

China

Rosa Rosania

Armenia

Gottfried Rudofsky

Germany

Victor Ruiz

Mexico

Vincenzo Russo

Italy

Ss Sa

Antigua And Barbuda

Zhi Xin Sahn

China

Ulrik Sartipy

Sweden

Lutz Schomburg

Germany

Jens Schreiber

Germany

Dfdsf Sdsf

Armenia

Claudine Seeliger

Germany

Ritsuko Seki

Japan

Norbert Senninger

Germany

Yang-Jo Seol

Korea South

Nasrullah Shah

Pakistan

Shuo Shi

China

Ching-Lin Shih

Taiwan

Yutaka Shimazu

Japan

Hugh Simpson

United Kingdom

Dongrong Situ

China

Dionisios Spyratos

Greece

Zaozhong Su

American Samoa

Yoshio Sumida

Japan

Azhar Supariwala

United States Of America

Jeff J Swensen

Australia

Jose Eduardo Tanus Dos Santos

Brazil

Xiaochun Teng

China

Andreas Thannheimer

Germany

Peter Tiidus

Canada

Naoto Tomita

Japan

Francesco Tonelli

Italy

Antoni Torres

Spain

Toshihiko Tsukada

Japan

Ilaria Uccella

Italy

Aydin Unal

Turkey

Martijn Van Griensven

Germany

Jeffery Vancem

United Kingdom

Seda Vatansever

Turkey

Pälvi Vento

Finland

Jean-Michel Vergnon

France

Sumita Verma

United Kingdom

Ross Vlahos

Australia

Guangyi Wang

China

Luhua Wang

China

Tamika Webb-Detiege

United States Of America

Doerte Wichmann

Germany

Amanda Williams

United Kingdom

Xiaoyan Xin

China

Cory Yamashita

Canada

Zifeng Yang

China

Felix Boon Bin Yap

Malaysia

Akihito Yokoyama

Japan

Toshinori Yoshida

Japan

Hui Yu

China

Ken Zafren

United States Of America

Alberto Zambelli

Italy

Paul Zarogoulidis

Greece

Mian Zeng

China

Qing Zhang

China

Sen Zhang

China

Yunguang Zhang

China

Changman Zhou

China

Hua Zhou

United States Of America

M. Zhou

United States Of America

Maryam Ziaei

Australia

